# Nutritional Status Associated with Metabolic Syndrome in Middle-School Children in the City of Montes Claros - MG, Brazil

**DOI:** 10.2478/hukin-2014-0094

**Published:** 2014-11-12

**Authors:** Igor Raineh Durães Cruz, Daniella Mota Mourão, Daniel Antunes Freitas, Andrey George Silva Souza, Alessandra Ribeiro Pereira, Felipe José Aidar, André Luiz Gomes Carneiro

**Affiliations:** 1 Integrated Colleges of Northern Minas Gerais – Funorte, Brazil.; 2 University of Tras-os-Montes and Alto Douro, Vila Real, Portugal.; 3 University President Antônio Carlos - UNIPAC, Uberlândia, MG, Brazil.; 4 Fire Department of the State of Minas Gerais, 5th Battalion Fire Department, Uberlândia, MG, Brazil.; 5 University Center Triangle - UNITRI, Uberlândia, MG, Brazil.

**Keywords:** dyslipidemia, metabolic syndrome, children, cross-sectional study, youth

## Abstract

The aim of this study was to investigate the association between nutritional status and prevalence of metabolic syndrome (MS) in middle-school students in the city of Montes Claros - MG. The sample consisted of 382 students, aged 10–16 years. Nutritional status was evaluated using the Body Mass Index (BMI). Metabolic syndrome (MS) was defined as the presence of two or more criteria in accordance with definition of the International Diabetes Federation. The overall prevalence of MS was 7.9%. 9.7% of students with MS were overweight and 72.4% were obese. Therefore, it can be inferred that carrying excess weight considerably increases the chances for a child to develop MS, and concomitantly increases the child’s risk for developing cardiovascular disease.

## Introduction

Metabolic syndrome (MS) is defined by the presence of risk factors such as high blood pressure, abdominal obesity, hypertriglyceridemia, low concentration of high density lipoprotein (HDL-c) and hyperglycemia (Eckel et al., 2005; Oda, 2012; Chiu et al., 2012). Prevalence of MS has increased among children, adolescents and adults, and is closely related to the presence of excess weight or obesity (Cruz and Goran, 2004; Zimmet et al., 2007).

No consensus has been reached in the literature regarding the diagnosis of MS in pediatric populations because blood pressure (BP), lipid profile and anthropometric values vary with age and maturity, requiring different cutoff points to account for variations in age, sex and other criteria (Damiani et al., 2011). However, the International Diabetes Federation (IDF) has established criteria for identifying SM in children and teenagers, dividing them into three groups: 6 to < 10 years old, 10 to 16 years old and > 16 years old (Zimmet et al., 2007; Damiani et al., 2011). In accordance with the criteria of the IDF, MS is a combination of abdominal obesity and one or more other risk factors (Zimmet et al., 2007).

Studies using the cutoff points established by the IDF indicate the presence of MS in 16.3% of children living in Malaysia (Zimmet et al., 2007), 4.6% of adolescents in Vietnam (Nguyen et al., 2011), 33% in Turkey (Sagun et al., 2011), and 39.7% of a municipal population in Brazil (Costa et al., 2012).

Investigations of various methodologies concur that MS is associated with cardiovascular diseases (CVD) regardless of age, and that excess weight or obesity is the most prevalent risk factor (Cobayashi et al., 2010; Armas et al., 2012). Thus, as the incidence of childhood obesity is rising, the prevalence of MS in this population is also increasing (Pacífico et al., 2011) and needs to be futher investigated in the Brazilian pediatric population (Silva et al., 2012; Stabelini et al., 2012).

The aim of this study was to investigate the association between nutritional status and prevalence of metabolic syndrome in middle-school children in the city of Montes Claros, MG, Brazil.

## Material and Methods

The sample was determined in two stages: 1) stratification of elementary school students (n = 53.032) and middle school students (n = 15.505), and 2) clustering of schools according to the four regions of the city: north (n = 10), south (n = 17), east (n = 15), west (n = 13), totaling 55 schools. Four schools (one from each region) were randomly drawn in order to minimize the logistical difficulties of displacement while acquiring a sufficient number of students to constitute the sample. At each of these randomly selected schools, all students between the ages of 10 and 16 years old who participated in physical education classes were invited to take part in the study. The calculation of the final sample was established with a margin of error of three percentage points and a confidence level of 95%, a design effect of 1.5 plus 10% for possible losses and / or refusals. Thus, 421 children were selected using the formula: n = (ZZ).p.q.N/e.e.(N-1)+ p.q.Z.Z, where Z = confidence interval, P = probability of being rejected 50%, q = probability of being chosen, 50% N= population and e = percentage of error ≤ 0.05 (Almeida and Freire, 2003; Cruz et al., 2014).

Of the 421 students initially selected, 15 students (12 boys and 3 girls) did not obtain the signature of a parent or guardian on the consent form; 4 children (3 boys and 1 girl) did not properly complete the questionnaire; 12 students (3 boys and 9 girls) did not complete the physical examination; and 8 male students were randomly excluded, reaching the final sample of 382 students distributed in the following regions: north (n = 62), south (n = 111), east (n = 105) and west (n = 104).

Informed consent to participate in the study was signed by a parent or guardian of every student included in the results of the study. The study was approved by the Institutional Review Board of the State University of Montes Claros (process no. 152.330/12). Participants were duly informed about the study and signed informed consent forms in accordance with Resolution 196/1996 of the Brazilian National Health Council, and in accordance with the ethical principles contained in the Declaration of Helsinki (1964, revised in 1975, revised in 1983, revised in 1989, revised in 1996 and 2000).

### Instruments

Socioeconomic status (CSE) of participants in the sample was attained by means of a structured questionnaire which took into account the educational background of parents, skin color of the children and family income. Based on this information, participants were classified into eight groups (A^1^, A^2^, B^1^, B^2^, C^1^, C^2^, D, E) and these eight groups were assigned to one of two categories of economic status: high economic status (A^1^, A^2^, B^1^) or low socioeconomic status (B^2^, C^1^, C^2^, D and E) (Fernandes et al., 2008).

### Variables of nutritional status and metabolic syndrome

Anthropometry: Body mass (BM) and height (H) were measured on a medical platform scale (Filizola, Brazil) with a weight capacity of 150 kg with precision to 100 g and a height capacity of 2 m with precision to 0.1 cm. The subject stood with his or her arms along the body and with his or her head positioned in the Frankfurt plane. Nutritional status was defined by the Body Mass Index (BMI) and classified as normal weight, overweight or obesity (Cole et al., 2000). Abdominal circumference (AC) was measured using a flexible metal anthropometric tape (Sanny SN 40-10, Brazil). The measurements were performed upon exhalation with the values on the measurement tape facing the evaluator and were determined in the horizontal plane. Males were evaluated at the plane just below the last rib and females evaluated at the level of the navel (Lohman et al., 1998).

*Blood pressure (SBP / DBP)* - was measured using a sphygmomanometer (BD, Brazil) and a Rappaport stethoscope (Premium, Brazil) that was previously tested and calibrated. The evaluation took place with the individual in a sitting position after 10 min of rest. The right arm supported at the heart level was used for the analysis. The first Korotkoff sound was considered for systolic blood pressure reading and the last for diastolic blood pressure. A five minute rest interval was given between the first and subsequent measurement. The average of the two measurements was calculated to minimize measurement biases (BGH, 2010).

*Blood samples* - blood samples for the biochemical tests were collected at Santa Clara Laboratory (Montes Claros, MG, Brazil) through venipuncture with disposable needles and syringes, with participants fasting for 12 hours. Approximately 10 ml of blood was collected in a test tube without anticoagulants. The test tubes were labeled with identification tags and stored in two coolers (®Termolar, Brazil) with a capacity of 200 tubes each. The tubes were placed in a centrifuge at 3500 rpm for 10 min and stored in a refrigerator at −20 ° C for later analysis. The concentrations of triglycerides (TG), high density cholesterol (HDL-C) and glucose (GLU) were determined by a calorimetric oxidase enzyme analyzer provided by Liquiform Diagnostic Kits ® (Labtest, Brazil).

The International Diabetes Federation (IDF) created age appropriate definitions and approaches to the treatment of SM for children and teenagers, dividing them into groups: 6 to < 10 years, 10 to 16 years and > 16 years. The IDF recommends that the diagnosis of MS should not be given to children under the age of 10, recommending that the child should be counseled about the need for weight loss and a change of lifestyle. However, at 10 years or older, the diagnosis should be given and should be based on the presence of abdominal obesity and the presence of two or more of the following syndromes: TG> 150 mg / dL, HDL <40 mg / dL, Glu> 100 mg / dL, SBP ≥ 130 and DBP ≥ 85 mmHg 20. For adolescents above the age of 16, the adult criteria are used (Damiani et al., 2011).

### Procedures

The subjects were evaluated by two physical education teachers with a minimum experience of 30 ratings. The Pearson values for intra-rater and inter-rater reliability were 0.975 and 0.967, respectively. Data collection occurred in four stages. In the first stage, the researcher presented the objectives of the project to the directors of the randomly selected schools. The school directors subsequently informed the physical education teachers about the study procedures. Individual participants were randomly drawn from the population of students who fulfilled the criteria for inclusion in the sample. In the second stage, the researcher presented the randomly selected students with the informed consent form to be completed by a parent or guardian and returned the next day. In the third stage, questionnaires were applied and anthropometric measurements were completed. In the fourth stage, the students came to the laboratory for blood sampling after fasting for 12 hours. The time between each step was a minimum interval of 24 hours.

### Statistics

The Statistical Package for Social Sciences (SPSS 20.0 for Windows ®) was used to analyze the data. Descriptive statistics were done through measures of central tendency mean ± standard deviation (M ± SD). We applied the chi-square test in order to associate prevalence of MS with nutritional status. The magnitude was calculated from the odds ratio (OR) with confidence intervals of 95% (95% CI).

## Results

Table 1 shows the description of the sample, categorized by nutritional status.

33.3% of male participants and 66.7% of female participants were found to be overweight and 34.5% of male participants and 65.5% of female participants were found to be obese. Students under the age of 13 were most affected by being overweight or obese. BM was greater among obese children. There were more overweight and obese children in the elementary school than in the middle school. Low income status was associated with children being overweight or obese.

Table 2 describes the criteria for diagnosis of MS, categorized by nutritional status.

The results show that being overweight corresponded with abnormal (greater than recommended) waist circumference (WC) in 12.5% of participants and other abnormal values, increasing the chances of diagnosis of MS, since the IDF uses the criterion of change in waist circumference combined with two criteria.

Students with obesity had a greater tendency towards abnormal values for the WC (OR = 15.55, CI: 5.43 to 44.51, p = 0.001), SBP (OR = 3.66, CI: 2, 65 to 5.05, p = 0.024), TG (OR = 7.29, CI: 1.32 to 40.07, p = 0.01) and GLU (OR = 3.66, CI: 2.65 −5.058, p = 0.024). The differences in risk factors between obese and normal weight children were significant, revealing a strong association between obesity and metabolic syndrome.

Table 3 describes prevalence of MS and its association with the nutritional status of schoolchildren in the city of Montes Claros.

The overall prevalence of MS was 7.9% in the sample of school children. However, only 0.7% of normal weight children and 9.7% of overweight children had MS, while 72.4% of obese children fulfilled the criteria for diagnosis of MS. This significant association (p = 0.001) demonstrates that the obese children were 24.37 times more likely to meet the criteria for diagnosis of MS (CI: 7.89 to 75.25).

## Discussion

The aim of this study was to investigate the association between nutritional status and prevalence of metabolic syndrome (MS) in middle-school children. The data from this study showed higher prevalence of excess weight or obesity among female students, indicating that girls are more likely to develop MS than boys. This corresponds to a similar study of 5913 participants in the United States in which females had a higher frequency of being overweight or obese when compared to males (Maty et al., 2008).

The relative values of height and weight are considered in the calculation of the BMI. In this way, a more comprehensive view considers that both height and age need to be considered when evaluating whether a child is overweight or obese. When a child is heavy for their height and age, slow and steady weight loss are recommended to reduce the BMI and minimize cardiovascular risk (Terres et al., 2006; Kassi et al., 2011).

In several studies it has been noted that education reduces or eliminates certain risk factors. For example, one study showed a significant negative association between lower levels of education and excess weight or obesity. This study relates that when parents have only five to eight years of schooling, the risk of their children being overweight or obese is 2.53 (CI: 1.32 to 4.85) times higher than amongst children whose parents have at least a high school education (Terres et al., 2006). Another study found that children tended to lose excess weight during high school when they were exposed to nutritional knowledge and tools to control weight (Barrington et al., 2010).

The present study found that non-white and low income children had higher incidences of being overweight or obese. Since non-white status is associated with lower incomes in Brazil, the similarity in these associations are related (Ribeiro et al., 2009). Similarly, a longitudinal study conducted with 29,146 Americans between 2 to 17 years showed more excess weight among black and Hispanic when compared with white children. The association between racial identity and nutritional status is considered to be a byproduct of unequal educational and vocational opportunities and cultural differences (Freedman et al., 2006). Numerous studies have associated lower socioeconomic status with obesity, hypertension and dyslipidemia (Kassi et al., 2011; Ribeiro et al., 2009).

Similar studies support a relationship between increased (abnormal) waist circumference (WC) and prevalence of children being overweight (12.5%) or obese (69%) and demonstrate that a high level of abdominal fat in childhood is related to insulin resistance, dyslipidemia and hypertension (Johnson et al., 2010). A recent study conducted in 991 schools in Paraná, Brazil showed that female students had a tendency toward abnormally high values for waist circumference and that this was related to the intake of excess sugar, limited physical inactivity, unhealthy family life styles and low socioeconomic status (Moraes et al., 2013).

BMI and WC are widely used as predictors of nutritional status, but the IDF also uses blood pressure as an essential criterion for diagnosis of MS (Reaven, 2006). Individuals with high BMI tend to have 3.6 and 2.7 times the risk of change in SBP and DBP, respectively (Ribeiro et al., 2006). Studies have demonstrated that elevated BP in childhood and adolescence is associated with anthropometric changes and a higher incidence of MS in adulthood (Ribeiro et al., 2006).

High concentrations of triglycerides present a picture of hypertriglyceridemia, which contributes to decreases in HDL-C and the production of LDL cholesterol particles. These changes result in an atherogenic lipoprotein profile congruent with an increased risk of cardiovascular disease (Moraes and Falcão, 2013; Reaven, 2006; Benmohammed et al., 2011). A case-control study of adolescents living in Malaysia indicated that being overweight or obese affects the odds of having high triglycerides by 12% and similarly resulted in a 2.5 times greater chance of developing MS (Costa et al., 2012).

Hyperglycemia was detected in 6.9% of children with obesity. It is possible that this relatively low prevalence could be inaccurate as the study did not investigate the use of hypeglycemic or diabetic medications among children in the sample. The IDF emphasizes the importance of measuring the fasting glucose as it is a more accurate measure of risk for MS (Zimmet et al., 2007). Another study suggests that hyperglycemia may not be concurrent with MS as it may occur later, when the function of the pancreas is challenged because of ongoing efforts to keep normal glucose levels through increased insulin production (Ferrannini and Iozzo, 2006). While it is not advisable to use glucose levels as a sole indicator of MS since studies of this relationship present a wide variety of criteria, the existent literature uniformly supports the presence of a positive association between increased glucose levels and higher prevalence of being overweight or obese (Moraes and Falcão, 2013; Papoutsakis et al., 2012).

In our study, 0.7% of normal weight, 9.7% of overweight and 72.4% of obese children met the criteria for MS, indicating that nutritional status plays a key role in its incidence. A case-control study with 402 children living in Malaysia found prevalence of MS of 16.3% among obese children and 5.3% among overweight children (Wee et al., 2011). Similarly, the relationship between MS and nutritional status was investigated in 693 high school students, finding prevalence of 4.6% and 11.8% among overweight and obese adolescents (Cobayashi et al., 2012). Our findings are also in agreement with the results of a cross-sectional survey conducted with 133 subjects of an endocrinology clinic that indicated the influence of obesity on the occurrence of MS, pointing out that one in five obese patients met three or more criteria for diagnosis of MS (Nguyen et al., 2010). In research conducted with hospitalized children and adolescents with leukemia in Saudia Arabia, MS was diagnosed in 7.1% of the total sample with higher incidences among overweight and obese subjects (Aldhafiri et al., 2012).

An important limitation of the present study was that as a cross-sectional study, it was only capable of describing the current status of the participating children and adolescents and, therefore, it was not capable of illuminating the causes and consequences of the problem

## Conclusion

In conclusion, nutritional status tends to be associated with MS among school children, with prevalence of 0.7% for normal weight students, 9.7% for overweight students and 72.4% for obese students in the study population, leading to the conclusion that obese participants were 24.37 times more likely to be diagnosed with MS, which implies a much higher degree of risk for cardiovascular disease.

## Practical Applications

This study confirmed an association between metabolic syndrome (MS) and being overweight or obese amongst school children in Montes Claros, Brazil. This indicates a great need for effective educational programs that promote an increase in physical activity and education in good nutrition. MS can be avoided by the adaption of better physical education programs in school, afterschool and on the weekends. This study indicates a need for public policies that incorporate health education in public programming and quality public and private education. A mere 30 minutes of physical activity three times a week could make a significant difference in the incidence of overweight and obese children, and, subsequently, reduce the risk of developing Metabolic Syndrome and consequent cardiovascular diseases.

## Figures and Tables

**Table 1:** Description of the sample categorized by nutritional status.

**VARIABLES**	**Nutricional Status**			**Total**

	**Normal weight**	**Overweight**	**Obese**	
**Gender**				
Male	117 (41,6%)	24 (33,3%)	10 (34,5%)	151 (39,5%)
Female	164 (58,4%)	48 (66,7%)	19 (65,5%)	231 (60,5%)
**Age**				
<13	121 (43,1%)	36 (50%)	16 (55,2%)	173 (45,3%)
≥13	160 (56,9%)	36 (50%)	13 (44,8%)	209 (54,7%)
**Weight (kg)**	44,44 ± 9,63	59,45 ± 10,83	72,79 ± 13,64	49,42 ± 13,51
**Height (m)**	1,55 ± 0,1	1,57 ± 0,09	1,54 ± 0,93	1,55 ± 0,1
**Education**				
1 st grade/ Elementary school	234 (83,3%)	67 (93,1%)	26 (89,7%)	327 (85,6%)
2 nd grade/High school	47 (16,7%)	5 (6,9%)	3 (10,3%)	55 (14,4%)
**Race**				
Non-white	233 (82,9%)	60 (83,3%)	26 (89,7%)	319 (83,5%)
White	48 (17,1%)	12 (16,7%)	3 (10,3%)	63 (16,5%)
**Socio-economic status**				
HSS	27 (9,6%)	8 (11,1%)	2 (6,9%)	37 (9,7%)
LSS	254 (90,4%)	64 (88,9%)	27 (93,1%)	345 (90,3%)

HSS: High Socioeconomic Status; LSS: Low Socioeconomic Status.

**Table 2:** Description of MS criteria met according to nutritional status.

**Variables**	**Nutritional Status**					***p***

	**Normal**	**Overweight**	**Obesity**	**OR**	**CI (95%)**	
**WC (cm)**						
Normal	281 (100%)	63 (87,5%)	9 (31%)			
Abnormal	--	9 (12,5%)	20 (69%)	15,55	(5,43–44,51)	0,000^*^
**SBP (mmHg)**						
Normal	281 (100%)	72 (100%)	27 (93,1%)			
Abnormal	--	--	2 (6,9%)	3,66	(2,65–5,05)	0,024^*^
**DBP (mmHg)**						
Normal	281 (100%)	72 (100%)	28 (96,6%)			
Abnormal	--	--	1 (3,4%)	3,57	(2,6–4,89)	0,113
**TG (mg/dL)**						
Normal	279 (99,3%)	70 (97,2%)	24 (82,8%)			
Abnormal	2 (0,7%)	2 (2,8%)	5 (17,2%)	7,29	(1,32–40,07)	0,01^*^
**HDL-c (mg/dL)**						
Normal	240 (85,4%)	56 (77,8%)	19 (65,5%)			
Abnormal	41 (14,6%)	16 (22,2%)	10 (34,5%)	1,84	(0,71–4,74)	0,202
**GLU (mg/dL)**						
Normal	278 (98,9%)	72 (100%)	27 (93,1%)			
Abnormal	3 (1,1%)	--	2 (6,9%)	3,66	(2,65–5,058)	0,024^*^

OR: odds ratio, CI: confidence interval. p: statistical significance from the chi-square test.

CA: waist circumference, SBP: systolic blood pressure, DBP: diastolic blood pressure, TG: triglycerides, HDL-C: High Density Cholesterol, GLU: glucose.

* p<0,05

**Table 3:** Description of prevalence (%) of MS with 95% confidence, according to nutritional status.

**Variables**	**Nutritional Status**					***p***

	**Normal**	**Overweight**	**Obesity**	**OR**	**CI (95%)**	
**Metabolic Syndrome**						
Absent	279 (99,3%)	65 (90,3%)	8 (27,6%)			
Present	2 (0,7%)	7 (9,7%)	21 (72,4%)	24,37	(7,89–75,25)	0,000^*^

OR: odds ratio, CI: confidence interval. p: statistical significance from the chi-square test.

* p<0,05
